# Hierarchical Identifier: Application to User Privacy Eavesdropping on Mobile Payment App

**DOI:** 10.3390/s19143052

**Published:** 2019-07-11

**Authors:** Yaru Wang, Ning Zheng, Ming Xu, Tong Qiao, Qiang Zhang, Feipeng Yan, Jian Xu

**Affiliations:** 1 School of Computer Science and Technology, Hangzhou Dianzi University, Hangzhou 310018, China; 2 School of Cyberspace, Hangzhou Dianzi University, Hangzhou 310018, China

**Keywords:** privacy security, mobile payment app, financial transaction action, traffic identification

## Abstract

Mobile payment apps have been widely-adopted, which brings great convenience to people’s lives. However, at the same time, user’s privacy is possibly eavesdropped and maliciously exploited by attackers. In this paper, we consider a possible way for an attacker to monitor people’s privacy on a mobile payment app, where the attacker aims to identify the user’s financial transactions at the trading stage via analyzing the encrypted network traffic. To achieve this goal, a hierarchical identification system is established, which can acquire users’ privacy information in three different manners. First, it identifies the mobile payment app from traffic data, then classifies specific actions on the mobile payment app, and finally, detects the detailed steps within the action. In our proposed system, we extract reliable features from the collected traffic data generated on the mobile payment app, then use a series of well-performing ensemble learning strategies to deal with three identification tasks. Compared with prior works, the experimental results demonstrate that our proposed hierarchical identification system performs better.

## 1. Introduction

With the rapid popularization of the smartphone and mobile E-commerce, mobile payment apps have advanced tremendously as an innovative payment style. Analysis company reports show that the total market value of China’s third-party mobile payment reached 47 trillion yuan in the fourth quarter of 2018 (https://www.analysys.cn/article/analysis/detail/20018972). Alipay serves as a typical mobile payment, winning the top name in mobile payment with a 53.71% market share (https://www.iimedia.cn/).

The problem of privacy and security has been concerned for both users and developers. To protect user’s privacy, most of the mobile payment apps normally implement end-to-end encryption techniques, which allows people to have full of trust in mobile payment apps. However, it still cannot close the door to an adversary who intends to monitor a user’s privacy. In a certain circumstance, an adversary may acquire a user’s information (such as what type of app is used, or what action is executed, or even specific steps of the action) by analyzing the encrypted traffic from the mobile payment app.

Traditional traffic analysis, such as the port-based method in Refs. [1,2], is simple and efficient. It can identify different application types by mapping well-known port numbers (e.g., HTTP uses port 80). However, some apps may not register a port number with IANAor use ports excluding the well-known port numbers. Most of researchers (see [3,4] for instance) focus on the payload-based technology, which classifies the traffic by inspecting the payload content of packets. However, due to the emergence of SSL/TLS encryption technology, the content of the payload cannot be obtained from the encrypted network traffic, that is payload-based technology fails in the face of encryption. To this end, different approaches [5,6,7,8] that combine statistical features with machine learning techniques have been proposed. More recently, the works in [9,10,11,12] by using different combinations of methods identified the types of network traffic.

The study of user action identification, capable of identifying specific user actions, has remained a hot topic in recent years. This type of analysis can help understand in-app behaviors and build behavioral profiles of mobile users. Thus, these information is able to be exploited for marketing studies and user reconnaissance within networks [13]. To our knowledge, most of the prior works aimed to identify communication actions (i.e., instant messaging actions or on email clients). However, few works have focused on financial transactions, such as money transfer or red packets (a red packet is an online money transfer with congratulatory messages via a mobile payment app). Recently, the work in [14] opened the way of identifying actions related to financial transactions. This work proposed a method based on supervised learning to identify red packets and transfer transactions from WeChat traffic. WeChat is a typical instant messaging app, supplying people with a messaging service. At the same time, it involves payment services. Nevertheless, the prior work was limited to the WeChat app, which cannot be extended to other payment apps for identifying financial actions. Besides, due to financial actions involving multiple transaction patterns, such as QR code (Quick Response Code) scanning (to pay bills), it is not enough to simply identify the red packets and transfer transactions. Furthermore, collecting the traffic data manually is an extraordinarily difficult and time-consuming process.

Hence, in this paper, we develop a hierarchical identification system to classify the widely-used financial transaction actions and steps of mobile payment apps via encrypted network traffic. The main contributions of this paper are listed as follows:

- We design a hierarchical identification system that first identifies the mobile payment app from traffic data, then classifies specific actions on the mobile payment app, and finally detects the detailed steps within the action.
- We implement an automated method to collect traffic and extract the features that are able to characterize user actions on the mobile payment app reliably.
- A method is proposed to deal with the ambiguous traffic (that is, similar traffic among different user actions) on the mobile payment app.
- It is proposed to consider a variety of ensemble learning strategies and evaluate their performance for the hierarchical identification tasks.

The remainder of this paper is organized as follows. Section 2 generalizes the related works. Section 3 characterizes the network traffic on mobile payment apps. Section 4 provides a comprehensive description of our proposed hierarchical identification system. In addition, the extensive results are illustrated in Section 5. Finally, Section 6 concludes this paper.

## 2. Related Work

In this paper, we mainly focus on identifying financial transactions on a mobile payment app by using the network traffic. Different from the traditional network traffic data from web browsers, most of the traffic from smartphones typically implements end-to-end encryption that aims to protect user’s privacy. Indeed, many works have analyzed the network traffic from smartphones in recent years, which can be roughly divided into two types: app identification and user action identification.

### 2.1. Mobile App Identification

Mobile app identification aims at identifying the network traffic belonging to a specific app. This type of research can help network administrators manage the network better. For instance, by identifying the specific mobile app, we can help administrators adjust the network equipment and parameters. Thus, it enables them to improve the Quality of Service (QoS) [13]. Besides, app identification is possibly exploited by an attacker to eavesdrop the user’s privacy. For example, an attacker can monitor the target victim’s (especially for a high profile user) traffic and then uncover what types of privacy-sensitive (such as dating and health) apps the victim is using by analyzing the network traffic. Thus, it inspires researchers to focus on the issue of privacy security and design more secure apps to prevent privacy leaks.

An early work in this field was proposed by Lee et al. [15]. The authors first made a comparison between the traditional network traffic and the smartphone traffic, where the smartphone apps were identified by using the payload signature. However, the result showed that only 15.37% of the flow was classified correctly. In addition, that method performed worse when faced with encrypted network traffic. Similarly, the work in [16] proposed a framework named NetworkProfiler, which identified Android apps by inspecting HTTP payloads. Furthermore, it was not robust to the encrypted network traffic.

The work in [17] proposed a fingerprinting scheme for devices by learning their traffic patterns through background activities. Supervised methods were used to train classifiers with features they collected. Wang et al. [18] designed a system for smartphone app identification by analyzing encrypted 802.11 frames. They collected data frames on target apps and classified the traffic data using a learning-based algorithm with extracted features.

Subsequently, an app identification system called Appscanner was proposed by Taylor et al. [9]. This system was able to collect the network traffic automatically and achieved on average 99% accuracy for identifying one single app. Besides, an extended work [12] provided a robustness analysis for Appscanner with mutative configurations. Meanwhile, a reinforced method was proposed to cope with the ambiguous traffic data. The experimental results demonstrated that it still obtained a remarkable performance despite the app version and the device being altered.

### 2.2. User Action Identification

The operations of apps performed by users unavoidably trigger network data transmission. These operations involve user–app interaction, leading to a particular operation presenting a fixed pattern (for instance, the traffic generated by browsing a personal homepage on Facebook is different from that generated by sending tweets on Twitter). Hence, the pattern can be used to identify a specific user action in network traffic. User action identification can be used to reveal some highly-sensitive information about a user’s behavioral preference. For instance, the information for dating privacy (e.g., frequent chatting and browsing the personal homepage on dating apps) or for individual health status (e.g., inquiry and consultation of disease information on health apps) can be eavesdropped. Therefore, it is urgent to strengthen privacy protection on user action.

Coull and Dyer et al. [19] first focused on analyzing the Apple iMessage service. They inferred the user operating system, fine-grained actions, and message languages through utilizing the size of the packet exchanged between the target smartphones and Apple servers. Conti et al. in [20,21] developed a framework that characterized the “shape” of traffic flows by leveraging the available information in IP and TCP headers. The authors mainly focused on two types of Android apps (i.e., social apps and the email apps) to identify the specific user actions. Followed by the works of Conti et al., Park and Kim et al. [22] proposed a framework by using a supervised machine learning method based on hierarchical clustering to classify user actions on the KakaoTalk app.

More recently, Fu et al. [10] designed a framework named CUMMAthat concentrated on identifying the service usage type through analyzing the network traffic on instant messaging apps, WeChat and WhatsApp. They extracted 54 statistical features for training and achieved great performance for testing. Saltaformaggio et al. [23] proposed a framework named Netscope that utilized the unsupervised learning method to identify user actions. The framework verified its effectiveness even though the network traffic was encrypted.

In general, most of the current works only focused on identifying communication actions (or non-financial transaction actions), such as sending text or pictures. Few works investigated financial transaction identification, such as transfer payment or QR code payment. Actually, there are varieties of traffic patterns between financial transactions and the other actions (see the details in Section 3). This might lead to the failure of current methods for financial action identification. To fill the gap, the work in [14] proposed a supervised learning mechanism to identify red packets and money transfer actions from the WeChat app. However, the work was limited to the WeChat app. That may result in a weakened performance for the identification tasks on a mobile payment app. Besides, for network traffic collection, they used an inefficient hand-crafted method.

Our work is different from the prior work on the following points:

- We propose a hierarchical identification system that is capable of dealing with three different identification tasks, from app identification to actions on the payment app, then to steps within the action.
- For traffic collection, we develop an automated method that can utilize scripts instead of the cumbersome hand-crafted method to collect the encrypted network traffic.
- We consider two widely-adopted apps, the WeChat app and the Alipay app. Compared with WeChat used by [14], Alipay mainly focuses on providing professional payment services, which indeed attracts a large amount of smartphone users. The reports show that Alipay occupies 53.71% of China’s mobile payment market share, which exceeds the 14.91% of WeChat.
- Last, but not least, we design an effective method to deal with the problem of ambiguous traffic, which makes our proposed identification system more robust.

## 3. Characterizing Network Traffic on the Mobile Payment App

In this section, we describe the types of user actions and the detailed steps within the action on a mobile payment app and characterize the network traffic on a payment app.

### 3.1. Description of User Actions and Steps

User actions on mobile payment apps are typically divided into financial and non-financial actions. The former actions usually include both (1) a payer making a payment and (2) a payee receiving the payment; the non-financial actions usually involve both (3) chatting with each other and (4) photo sharing. Table 1 shows eight actions commonly executed by users on a mobile payment app, such as Alipay. The action types are shown in the leftmost column, the detailed steps’ description within each action in the middle column, as well as its corresponding label in the rightmost column. The first two actions represent non-financial ones, while the remaining represent financial actions. As Table 1 illustrates, the non-financial actions only consist of one step within one action. However, the financial actions (except the transfer receipt action) generally consist of at least two steps. For instance, the transfer payment action includes three steps: First, users enter the payment page by clicking the button for fund transfer, and then, the specific amount of transaction money is confirmed; finally, the personal password is input to finish this action.

### 3.2. Traffic Characteristics on a Payment App

**Figure 1:**
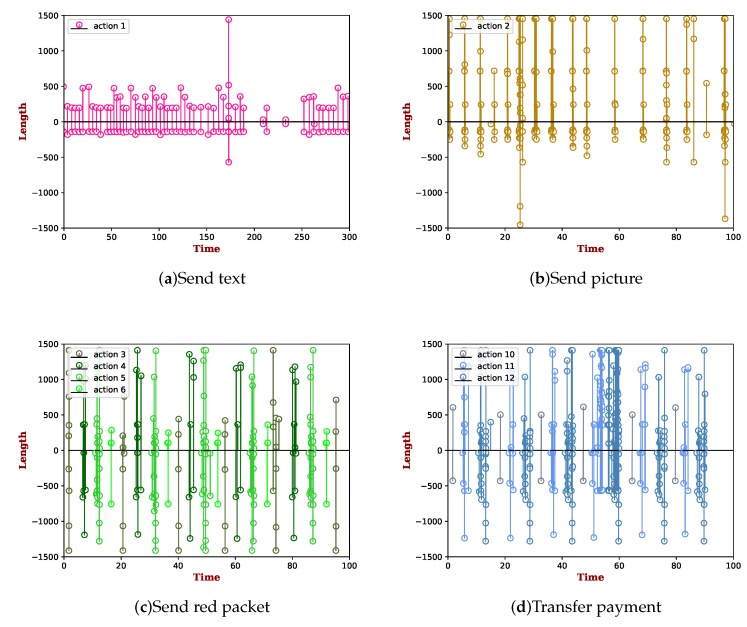
Packet time series distribution of user actions on the Alipay app.

Figure 1 represents the packet length of user actions on the Alipay app. The packet length is from two directions. One direction is incoming (i.e., send packets from the server port to the user port) with negative values, and the other is outgoing with positive values (i.e., send packets from the user port to the server port). In order to better characterize the traffic data, the two typical non-financial actions (see Figure 1a,b) were chosen to compare. For mobile payment apps, they not only involve the financial actions, but also the non-financial ones. The traffic data of financial actions are illustrated in Figure 1c,d. We selected the typical financial actions (i.e., send red packet and transfer payment) to exemplify. The discriminative characteristics are generally summarized as follows:

- **Different steps’ distribution**: As the aforementioned illustration in Table 1, unlike Actions 1 and 2, the financial actions tend to involve many steps. It is worth noting that there is a very discriminative pattern in each step. For instance, Figure 1c shows the process of sending a red packet that consists of four steps within the action, where the different colors represent different steps. As we can see, in the former two steps (i.e., Steps 3 and 4), before the red packet is sent out, only a small amount of packet exchanges is triggered. However, when a user inputs the password to pay (i.e., Step 5), it will carry plenty of information to the server, such as the amount of money, user password, and fingerprint. Thus, it generates many more packet exchanges than the former two steps. This unique characteristic indeed implies that it is feasible to identify each step within an action by learning its corresponding traffic patterns.
- **Different length distribution**: Indeed, there is a different length distribution between financial actions and non-financial actions. For example, sending a text does not cost much time and has a small data size (i.e., the packet lengths usually fall in the range of 0–500 bytes). However, for the actions of transferring a payment, they commonly involve packets with very different lengths. Especially for the third step (i.e., Step 12) within the action, the packet lengths usually fluctuate in the range of 0–1500 bytes, where in there exist not only small packets, but also fully-loaded packets (i.e., packet lengths close to 1500 bytes) and other different lengths of packets. The distribution of packet lengths has a larger variance than that of the non-financial ones.
- **Frequent host interaction**: Due to the financial actions on a mobile payment app involving many steps, each step has specific functions and accomplishes the different tasks of money flow. Host interactions from a user port to the different server ports appear frequently. Thus, plenty of TCP handshake packets are generated in the process of user operations. However, for the non-financial actions, these unique characteristics do not exist.

## 4. The Overview of the System Framework

**Figure 2:**
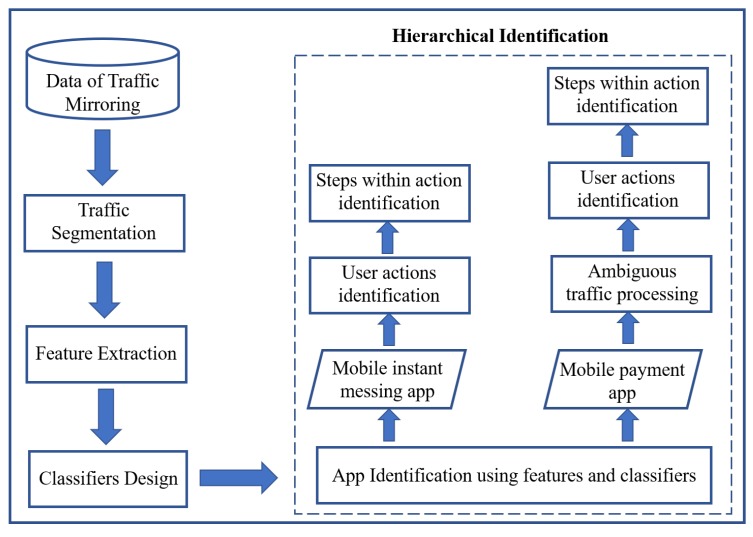
Hierarchical identification system.

Figure 2 illustrates the framework of our hierarchical system. The five main modules are listed as follows: (1) traffic mirroring; (2) traffic segmentation; (3) feature extraction; (4) classifiers design; and (5) hierarchical identification. In the hierarchical identification system, we first identify the types of app (i.e., instant messaging app or mobile payment app (In this paper, we exemplify two typical kinds of apps related to the financial transaction actions. In fact, our proposed system can be smoothly extended to other types of apps.)) from the mixed traffic data by using the ensemble learning classifier, then identify actions and steps, respectively. It is worth noting that the mixed traffic data refer to the traffic generated from six different apps (WeChat app, Alipay app, and other widely-used apps).

### 4.1. Traffic Mirroring

**Figure 3:**
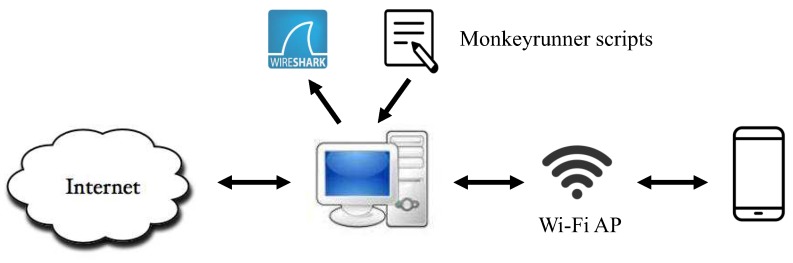
Configuration environment of traffic mirroring.

The configuration of collecting network traffic is illustrated in Figure 3. In this configuration, we established the Wi-Fi access point, which can provide a network environment to the target smartphone. To generate the network traffic, we implemented an automated method, exploiting scripts to simulate user actions on the mobile payment app. These scripts were compiled based on Monkeyrunner, which is a tool that enables programmers to control real devices with their provided APIs. Meanwhile, the action types were labeled by recording the time when a single step of an action was executed, then we used a free packet analyzer, Wireshark, to capture the network traffic. Then, both the network traffic and its corresponding label were stored in our data repository. Additionally, to minimize the “traffic noise”, we only captured the network traffic generated by the target app.

Compared with the works of [10,14], we implemented a method that automatically collects the network traffic on the mobile payment app instead of hand-crafted operation. Thus, the efficiency can be remarkably improved at the traffic collection stage.

### 4.2. Traffic Segmentation

We first introduce the basic terminologies that are necessary for understanding our methodology:

- **Burst**: A burst is a group network traffic that contains many packets where each time interval is smaller than a given time threshold (we define it as the *burst threshold*), regardless of their source or destination IP address.
- **Burst threshold**: The burst threshold is time limited, which can be used to terminate the burst when it does not receive any packets within a threshold time period. For instance, if the time interval between two adjacent packets is larger than a given threshold time, they will be segmented into two different bursts.

We used the burst threshold to segment the network traffic. Inspired by Ref. [14], we utilized a small burst threshold (i.e., 1.25 s), which can segment traffic data into a sequence of short time blocks. Thus, not only a single user action can be segmented, but each step within an action also can be segmented.

### 4.3. Feature Extraction

After segmenting the traffic into bursts, we extracted feature vectors, which consist of the following statistical features.

- **Overall statistics of the packet length**: Extracting the overall statistics of the packet length can describe the basic properties of the packet length distribution. In each burst, the first order and the second order statistics of the packet length were extracted as features, including the sum, mean, standard deviation, skewness, and kurtosis.
- **Range statistics of the packet length**: The range statistics of the packet length is the total number of packets whose length falls in a specific range. We first divided the total length range (i.e., 0∼1500 bytes) into several equalized sub-ranges. Then, for each sub-range, the features were obtained by calculating the total number of packets whose length fell in this range. By using the features, the influence of noise caused by the small fluctuations of packet length can be eliminated.
- **Flow statistic**: We utilized the flow statistical features to describe flows’ distribution in each burst. A flow is a sequence of packets within a burst with the same destination IP address and port number, that is all packets within a flow will either be going to or coming from the same IP address/port. In this context, we divided the burst into different flows and summed the flows within each burst.
- **Incoming and outgoing statistics**: The packets are exchanged in two directions during a TCP session, including the incoming and outgoing direction. Different directions have different traffic patterns. For instance, the total transported bytes at the transfer payment stage are different than at the transfer receipt stage. We collected the incoming and outgoing statistics to represent the different characteristics of traffic patterns from different directions.

### 4.4. Classifying Algorithm Design

In this paper, four ensemble learning algorithms are considered. It is proposed to analyze the performance for each algorithm. Next, let us describe the different ensemble algorithms in detail.

#### 4.4.1. RF

RF (Random Forest) is an ensemble learning algorithm that uses multiple decision trees to train and predict samples. It constructs a multitude of decision trees at the training stage, and at the prediction stage. It outputs the predicted class label that is the mode of classes of the individual trees [24,25]. For large-scale heterogeneous data, RF shows its great efficiency and simplicity. Depending on the parallelized training stage, it can quickly produce classifiers with high accuracy. Furthermore, it can also deal with the issues of overfitting at the stage of training decision trees.

#### 4.4.2. AdaBoost

AdaBoost (Adaptive Boosting) is a machine learning meta-algorithm that combines the decisions of different learning algorithms (weak learners) to improve performance [26]. Unlike RF, AdaBoost assigns weight values to the corresponding base learners at the iterative training phase. At the testing phase, it outputs a decision class label by combining the weighted sum of base learners. The information gathered at each stage of the AdaBoost algorithm about the relative “hardness” of each training sample is fed into the tree-growing algorithm, thus enabling them to focus on harder to classify instances.

#### 4.4.3. GBDT

GBDT (Gradient Boosting Decision Tree) is a technique that builds a prediction model via an ensemble of decision tree models [27]. Like AdaBoost, it produces a model in a stage-wise fashion. However, the differences are that the gradient descent method is chosen to ensure the best performance in the phase of iteration.

#### 4.4.4. XGBoost

In this paper, we also considered an innovative ensemble learning algorithm, XGBoost (eXtreme Gradient Boosting). XGBoost is a highly-scalable machine learning system for gradient boosting [28]. It improves machine learning algorithms under the gradient boosting framework by dealing with the bias-variance tradeoff even more carefully, directly leading to the improvement of prediction accuracy. Furthermore, it can automatically use CPU multi-threads for parallel computing.

In this paper, an exhaustive search on a set of hyperparameters (with 10-fold cross-validation) was used to optimize these parameters. For the RF classifier, we finally set the parameters *criterion = gini, n_estimators = 100, max_features = none, max_depth = none, min_samples_split = 4*. For the AdaBoost classifier, we used the parameters *algorithm = “SAMME”, n_estimators = 100, learning_rate = 0.1*. For the GBDT classifier, we set the parameters *n_estimators = 100, max_depth = 7, learning_rate = 0.1*. For the XGBoost classifier, we used the parameters *n_estimators = 300, max_depth = 7, learning_rate = 0.1, subsample = 0.8.*

### 4.5. Hierarchical Identification

In the real scenario, attackers possibly would like first to understand the action performed on which smartphone app before knowing the types of specific actions. To this end, the adversary can further improve the accuracy of targeting the victim and achieve the expected attack goal within a very short time. Besides, financial actions usually involve many different steps. Especially for the payment steps (i.e., input the password to pay), the traffic involves plenty of privacy information such as the user’s password and fingerprint. By classifying each specific step within action, the attacker is able to intercept the corresponding traffic and then exploit it to make further analysis, such as mining a user’s privacy information.

To deal with the proposed problems, we exploited a hierarchical identification strategy that identified the traffic data in three different ways. In particular, we first identified the payment app from mixed traffic data. Then, we identified different user actions on the mobile payment app. Finally, we identified the specific steps within each action. The following lists three identification methods.

#### 4.5.1. App Identification

In this paper, we intended to identify two typical apps (WeChat and Alipay) from the mixed network traffic. As a typical mobile payment app, Alipay has professional payment services, while providing a simple instant messaging service. Conversely, WeChat is a typical mobile messaging app, supplying people with fast and efficient messaging services. Meanwhile, it involves payment services similar to Alipay. That is, WeChat does not only have financial actions, but also non-financial ones.

For app identification, we first extracted features (the features we introduced in Section 4.3) from the training set, which was used to train each sub-classifier. During the stage of testing, the predicted result for unknown mixed traffic data can be obtained by combining the predicted results of different individual sub-classifiers within the ensemble learning algorithm. Finally, the mixed traffic was identified (labeled as Alipay app or WeChat app for instance). Note that, except for Alipay and the WeChat app, it was proposed to select the four other widely-used apps (i.e., Weibo, Tik Tok, Weishi, and Taobao) to generate the mixed traffic data.

#### 4.5.2. User Action Identification

When the app was identified, we further identified the specific actions. We characterized the distinguishable patterns of traffic data between financial and non-financial actions in Section 3. For action identification on the WeChat app, we directly extracted features to train ensemble learning models. Unfortunately, different from WeChat app, the traffic data of actions on Alipay app involved different kinds of ambiguous traffic that behaved similar to each other among different user actions. This possibly hindered the modeling process. The following lists two types of ambiguous traffic on Alipay:

- **Background traffic**: In the process of user operation, many small-scale packets whose length ranged from 0 to 35 bytes (it might be heartbeat packets) were generated.
- **Confusion flow**: Since the distribution of the traffic pattern had a large variance, it confusion flows caused by common SSL handshakes would appear. These kinds of network traffic are usually similar among the overall types of actions. The confusion flows would frustrate the modeling process because the machine learning algorithms would be given contradictory training examples.

To this end, we directly filtered out the background traffic whose lengths ranged from 0 to 35 bytes. Furthermore, to reduce the effects of confusion flow caused by SSL handshakes, we used the Dynamic Time Warping (DTW) [29] algorithm, which is used to measure the similarity between two temporal sequences of unequal length. Specifically, given a burst instance, which consisted of the flows {$f_{i1},f_{i2},...,f_{in}$}, before processing, the template of confusion flows was elected from traffic data, which consisted of {$f_{t1},f_{t2},...,f_{tk}$}, we used the DTW algorithm (denoted as the function $F_{DTW}$) to calculate the distances of the flow between the template and burst instance, that is,

(1) $$dist\left(f_{ia},f_{tb}\right)=F_{DTW}\left(f_{ia},f_{tb}\right)\;$$

a∈(1,...,n),b∈(1,...,k)

then the distance matrix was obtained as $\left\{D_{1},D_{2},...,D_{n}\right\}$, where $D_{i}$ is the distance of a single flow within a burst instance to the template members that contain the column vector as ${\left\{d_{1},d_{2},...,d_{k}\right\}}^{T}$. For each $D_{i}$, we selected the minimum value among the overall vector members, which is denoted as,

(2) $$d_{min}=\min\left(d_{j}\right),j\in \left(1,...,k\right)\;$$

We set a distance threshold (i.e., the value of the threshold was 500), which was a criterion for the judgment of confusion flow. If the minimum distance of flow within burst was larger than the threshold, it would be regarded as a confusion flow. After removing, we extracted feature vectors for each burst and fed them into the ensemble learning algorithm, respectively. Then, the corresponding labels of actions on Alipay were identified from the testing set.

#### 4.5.3. Step Identification within an Action

We speculated that attackers would make more fine-grained privacy eavesdropping. Therefore, we intended to take more fine-grained identification. In this subsection, we focus on identifying each step within each action. Once the traffic data were categorized as specific user actions on WeChat or Alipay, we immediately re-labeled the dataset and classified them into different steps.

## 5. Evaluation

In this section, we evaluate the performance of our proposed hierarchical identification system. In this context, the evaluation of the privacy eavesdropping refers to verifying the effectiveness of the proposed hierarchical system for identifying the mobile payment app. For each identification task in our system, we first extracted features from traffic data and fed them into the ensemble learning algorithm, then used the produced prediction models to complete the identification task via using unknown encrypted traffic data. Moreover, in order to verify the relevance of our system, each prediction model was evaluated using 10-fold cross-validation with instances drawn from each subset of the dataset.

### 5.1. Data Description

For data collection, we used the proposed automated scripts to simulate user actions, while Wireshark was used to capture the generated traffic data. The scripts ran on a Samsung smartphone (Samsung Galaxy S II) with the Android operating system (Version 6.6.1). For hierarchical identification including the app, action, and step, we used Alipay, WeChat, and four other widely-adopted apps (Weibo, Tik Tok, Weishi, and Taobao) to generate mixed traffic data. Table 2 illustrates the total statistics of network traffic generated on these six different apps. For simplicity, it was proposed to only identify the traffic data generated for Alipay or the WeChat app. Table 3 shows the detailed data statistics of different actions on Alipay and the WeChat app. Each action in an app was executed about 150 times in 120 min.

### 5.2. Evaluation Metrics

In this subsection, four evaluation metrics used to evaluate our system performance are listed as follows:

- Accuracy denotes the percentage of both true positives and true negatives among the total number. The formula is denoted as:
  (3) $$\mathrm{accuracy}=\frac{TP+TN}{TP+FP+TN+FN}\;$$
  where TP and TN represent the true positives and true negatives for all classes and FP and FN denote the false positives and the false negatives.
- Recall is the percentage of the number of instances correctly classified among the number of all positives. Its formulation is given by:
  (4) $$\mathrm{recall}=\frac{TP}{TP+FN}.\;$$
- Precision is the percentage of true results (correctly positive instances classified) among the total number of positive instances classified, described as:
  (5) $$\mathrm{precision}=\frac{TP}{TP+FP}.\;$$
- F1 is the harmonic mean of precision and recall. It is given by the following formulation:
  (6) $$F1=\frac{2\times P\times R}{P+R}\;$$

where *P* and *R* respectively denote the precision and recall.

### 5.3. Results on App Identification

We first aimed at identifying the Alipay app and WeChat app from mixed traffic data. Table 4 shows the results for app identification using different evaluation metrics (i.e., accuracy, recall, precision, F1). As we can see, the use of ensemble learning algorithms (i.e., RF, AdaBoost, GBDT, XGBoost) can bring high overall accuracies (i.e., the accuracy of Alipay was larger than 0.98; WeChat was larger than 0.96). Especially when we used the AdaBoost algorithm to deal with the task of Alipay app identification, the accuracy could achieve 0.991, with a recall of 0.995, a precision of 0.986, and an F1 of 0.988. The nearly-perfect results indicated that we were almost able to identify the Alipay app perfectly from the mixed traffic data. Meanwhile, it is worth noting that the results on the Alipay app were slightly higher than the WeChat app. This is because the ambiguous traffic on Alipay might increase the differences between the apps.

### 5.4. Results on Action Identification

In this subsection, we mainly focus on the performance evaluation of user action identification on different apps, such as Alipay or WeChat. The detailed empirical analysis will be extended in the following.

#### 5.4.1. Results on Alipay Data

For the data on Alipay app, we trained the prediction models with features extracted from the training set after removing ambiguous traffic, and then, the identification performance was measured. Table 5 shows the comparison results of our method with the prior state-of-the-art method [14] by using four ensemble learning algorithms. Accordingly, our proposed system obtained the best performance with the AdaBoost algorithms. It had a precision of 0.975, recall of 0.974, accuracy of 0.965, and F1 of 0.974. It is obvious that our system can effectively identify different user actions (i.e., eight types of actions; see Table 1) on the Alipay app. In particular, the results of our proposed method with the AdaBoost algorithm provided an improvement over the prior method, for instance +0.045 (precision on Alipay data) and +0.059 (recall on Alipay data). The very promising results directly indicate that the adoption of ambiguous traffic removal (see details in Section 4.5) is feasible, and also, the features we extracted can more accurately characterize the network traffic of user actions on the mobile payment app.

**Figure 4:**
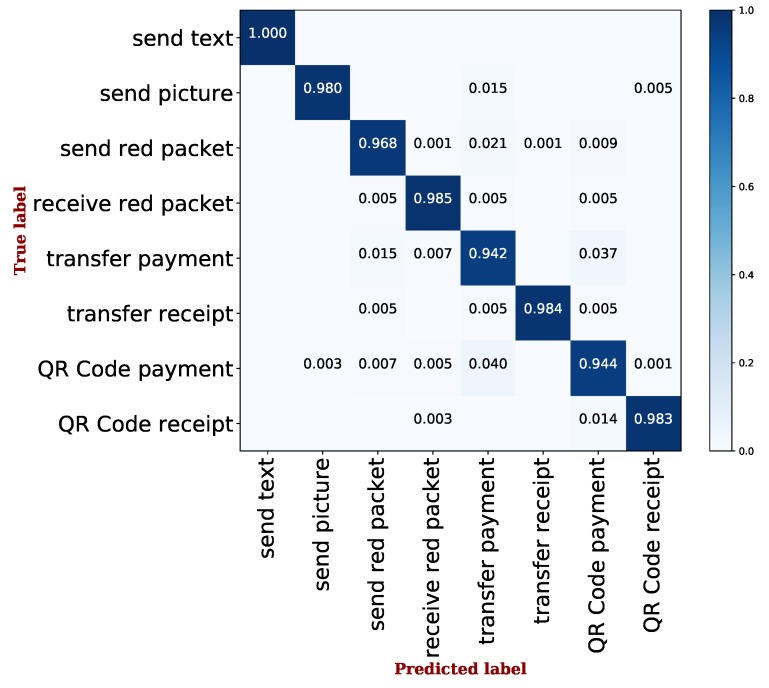
The confusion matrix on action identification using the AdaBoost algorithm.

Furthermore, Figure 4 shows the confusion matrix of the results from the best performing AdaBoost algorithm. In the confusion matrix, the horizontal axis represents the action types in the predicted label from the output of classifiers, while the vertical axis represents the one in a true label. In particular, each cell along the main diagonal describes the correct rate of one single action identification, where the darker cell represents the higher correct identification rate. Accordingly, the results nearly approached a 0.98 correct rate for most single action identification. However, for some financial actions, such as send red packet, transfer payment, and QR code payment, the correct rate was a little lower than other actions. This is because this type of action includes four complex steps, where each step generates many difficult to remove ambiguous traffic. Probably, that led to the slight increment of the misidentified rate. Nevertheless, the overall results still demonstrate that our system can correctly and effectively identify different user actions on the Alipay app.

#### 5.4.2. Results on WeChat Data

After evaluating the results of action identification on Alipay data, let us show the performance of identification on WeChat data. As Table 6 illustrates, our proposed method based on the AdaBoost algorithm obtained 0.987 accuracy, which is the most outstanding performance compared to the others. Nevertheless, the accuracy of the other classifiers also achieved above 0.97. Compared with the prior method [14], the performance of our method has slightly increased, where the improvement ranged from +0.002 (accuracy on WeChat data) to +0.006 (recall on WeChat data). Even though the identification accuracy was comparable with the prior work, our proposed scheme indeed extended that work, which can effectively complete the identification task on WeChat and Alipay, both of which occupy 92.65% of the total mobile payment market in China. Besides, our system can automatically collect payment-related data, which avoids the time consumption at the stage of traffic mirroring (see Section 4.1).

### 5.5. Results on Step Identification

#### 5.5.1. Results on Alipay Data

Table 7 reports the overall comparison results of step identification by using the ensemble learning algorithm on Alipay data. Overall, the best results were still from our proposed system adopting by the AdaBoost algorithm with the highest accuracy of 0.939. It outperformed the remaining classifiers since AdaBoost uses aggregated weak classifiers, which can reduce more bias. To our knowledge, AdaBoost comprehensively considers the suitable weight of each base learner, resulting in the generalization ability being able to be remarkably improved. Furthermore, similar to the results of action identification, step identification on Alipay data remarkably outperformed the prior method [14], where the improvement arrived at +0.120 accuracy and +0.142 recall.

**Figure 5:**
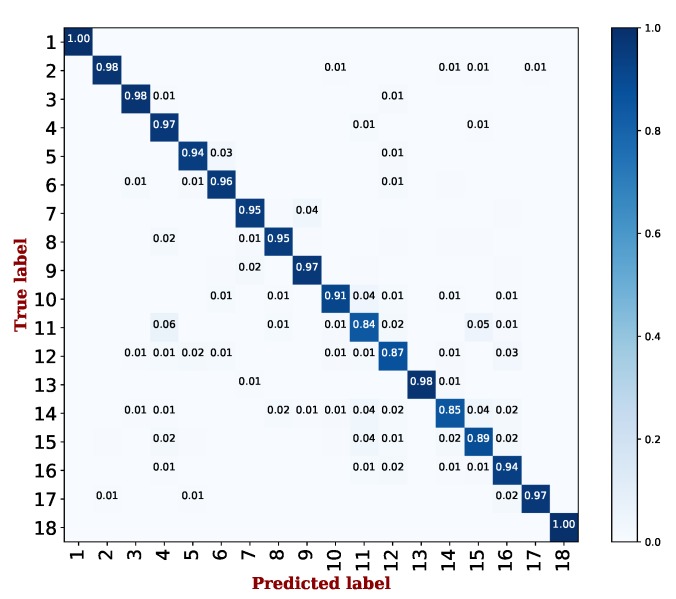
The confusion matrix on step identification using the AdaBoost algorithm, where each step index refers to Table 1.

Figure 5 also shows the confusion matrix for the best performing AdaBoost algorithm. For simplicity, we report the step index (i.e., 1∼18) on the axis as we have mentioned in Table 1. Similar to action identification, the vertical axis represents the true labels, and the horizontal axis represents the predicted labels. As Figure 5 illustrates, most of the steps can obtain accuracy above 0.95, the exceptions being for step identification within transfer payment (Steps 10∼12) and QR code payment (Step 14∼16). The results empirically verified that our method was able to identify the different steps within an action effectively on the Alipay app.

#### 5.5.2. Results on WeChat Data

Next, it was proposed to evaluate the performance of step identification on WeChat data. Table 8 illustrates the comparison results between the proposed learning classifiers. As we can see, the results illustrate that the AdaBoost algorithm was able to provide the highest identification rate (including four evaluation metrics) among the others. The findings suggest that the proposed hierarchical system can accurately estimate the steps within an action the on WeChat app. Meanwhile, compared with the prior version of [14], the improvement of the best algorithm, in terms of accuracy and recall, was respectively +0.006 and +0.029.

In fact, the extensive experimental results have verified that our system can effectively deal with three different identification tasks, including app, action, and step identification. Furthermore, our proposed hierarchical identification system relevantly performed well (especially adopted by the AdaBoost algorithm) on both the instant messaging app (WeChat app) and the mobile payment app (Alipay app).

### 5.6. Extended Experiments

In this paper, we proposed a hierarchical system that can deal with three identification tasks (i.e., app identification, action identification, and step identification). In order to demonstrate the general usability of our method, we extended our system to the UnionPay app. UnionPay as a banking unified app (also a mobile payment app) was established by UnionPay and commercial banks. The report (http://corporate.unionpay.com/infonewsCenter/infoCompanyNews/file_145773919.html) showed that the number of users on the UnionPay app had exceeded 100 million by November 2018.

In our system, we first identified the UnionPay app from the mixed traffic data. Next, we report the performance of our system. Table 9 shows the performance of UnionPay app identification. As we can see, the four ensemble learning strategies obtained high overall accuracy which was larger than 0.97. Especially when we used the AdaBoost or XGBoost algorithm, the accuracy could achieve 0.994. Moreover, these two algorithms also achieved good performance under the other evaluation metrics.

After identifying the UnionPay app, we identified the types of actions on the UnionPay app. For the traffic data of actions on the UnionPay app, it also involved the ambiguous traffic, which was same as the Alipay app. To this end, we also dealt with the ambiguous traffic with the aforementioned methods (see Section 4). The results of user action identification on the UnionPay app are shown in Table 10. As we can see, GBDT and XGBoost can obtain around 0.902 accuracy, which is the most outstanding performance compared to the other ensemble classifiers. The accuracies of the other algorithms also achieved above 0.89. On the whole, the four ensemble learning strategies performed well on the action identification task on the UnionPay app. However, all of them had a slightly weaker performance than the task on WeChat and Alipay. The traffic generated on the transfer receipt action was almost identical to the traffic on the QR code receipt action. Thus, that possibly led to a slight increase in the misidentified rate.

Last, we evaluated the performance on the UnionPay step identification. Table 11 illustrates the comparative performance between our ensemble learning classifiers in step identification on the UnionPay app. As we can see, the best result was an accuracy of 0.808 by adopting the RF algorithm. The findings suggest that our system can accurately estimate each step within actions on the UnionPay app.

## 6. Conclusions

In this paper, a hierarchical identification system was proposed that was able to analyze network traffic generated on a mobile payment app and to eavesdrop user’s privacy in three different manners, referring to app identification, user action identification, and step identification. The experimental results demonstrated that our hierarchical system is an effective tool for an attacker who can leverage it to eavesdrop the user’s privacy information on a mobile payment app. Meanwhile, our contribution will inspire more researchers to focus on the issue of privacy security and choose more effective defense strategies for user’s privacy protection on mobile payment apps.

In future studies, we will continue to focus on this research and aim at further extending our identification system. We intend to consider more mobile payment apps (such as PayPal, Samsung Pay, Google Pay). Besides, we hope to further understand the privacy security issues on the mobile payment app.

## Figures and Tables

**Table 1:** Illustration of both user actions and steps within an action.

Action Type	Step Name	Step Index
**Send text**	(i) send text to user’s friends	1
**Send picture**	(i) send picture to user’s friends	2
**Send red packet**	(i) click the button for the red packet (ii) fill in the fund amount, and click the pay button (iii) input the password to pay (iv) receive the open red packet	3 4 5 6
**Receive red packet**	(i) receive a red packet (ii) click the red packet (iii) open the red packet	7 8 9
**Transfer payment**	(i) click the button for fund transfer (ii) fill in the fund amount, and click the pay button (iii) input the password to pay	10 11 12
**Transfer receipt**	(i) receive a fund transfer	13
**QR code payment**	(i) scan the payment QR code (ii) fill in the fund amount and click the pay button iii) input the password to pay	14 15 16
**QR Code receipt**	(i) scan successfully (ii) receive a QR payment	17 18

**Table 2:** Traffic data statistics of the apps.

Number	Name of App	Packets	Bytes
**1**	Alipay	296.5k	264,207k
**2**	WeChat	939.8k	846,403k
**3**	Tik Tok	240.1k	299,494k
**4**	Weibo	47.8k	40,070k
**5**	Taobao	103.1k	118,331k
**6**	Weishi	846.4K	873,052k

**Table 3:** Traffic data statistics of user actions on the Alipay app and the WeChat app.

Action Types	Alipay				WeChat			
	Duration	Numbers	Packets	Bytes	Duration	Numbers	Packets	Bytes
**Send text**	1 h	198	0.7k	152k	1 h	192	0.8k	125k
**Send picture**	2 h	195	233.2k	237,568k	2 h	193	870.1k	827,444k
**Send red packet**	2 h	169	17.3k	7524k	2 h	271	17.1k	5153k
**Receive red packet**	2 h	205	3.2K	2104k	2 h	320	8.5k	2458k
**Transfer payment**	2 h	255	16.5k	6915k	2 h	144	10.2k	2772k
**Transfer receipt**	2 h	183	2.7k	862k	2 h	218	13.0k	3192k
**QR code payment**	2 h	248	19.7k	8255k	2 h	253	18.7k	4793k
**QR code receipt**	2 h	172	3.2k	827k	2 h	110	1.4k	466k

**Table 4:** Results on the Alipay app and the WeChat app identification.

Algorithms	Alipay				WeChat			
	Accuracy	Recall	Precision	F1	Accuracy	Recall	Precision	F1
**RF**	0.986	0.990	0.980	0.984	0.968	0.951	0.986	0.960
**AdaBoost**	**0.991**	0.995	0.986	0.988	**0.972**	0.946	0.997	0.964
**GBDT**	0.988	0.989	0.992	0.983	0.971	0.951	0.990	0.963
**XGBoost**	0.988	0.993	0.982	0.987	0.964	0.936	0.990	0.955

**Table 5:** Results of action identification on the Alipay app.

Algorithms	Our Method				Prior Method [14]			
	Accuracy	Recall	Precision	F1	Accuracy	Recall	Precision	F1
**RF**	0.961	0.968	0.971	0.969	0.901	0.906	0.916	0.909
**AdaBoost**	**0.965** **(+0.056)**	**0.974** **(+0.059)**	**0.975** **(+0.045)**	**0.974** **(+0.056)**	0.909	0.915	0.930	0.918
**GBDT**	0.960	0.968	0.971	0.969	0.906	0.910	0.920	0.913
**XGBoost**	0.963	0.969	0.971	0.970	0.910	0.914	0.933	0.922

**Table 6:** Results of action identification on the WeChat app.

Algorithms	Our Method				Prior Method [14]			
	Accuracy	Recall	Precision	F1	Accuracy	Recall	Precision	F1
**RF**	0.978	0.979	0.980	0.979	0.976	0.975	0.978	0.976
**AdaBoost**	**0.987** **(+0.002)**	**0.987** **(+0.006)**	**0.988** **(+0.002)**	**0.986** **(+0.003)**	0.985	0.981	0.986	0.983
**GBDT**	0.982	0.980	0.987	0.983	0.980	0.980	0.984	0.981
**XGBoost**	0.986	0.984	0.986	0.985	0.983	0.982	0.985	0.983

**Table 7:** Results of step identification within an action on the Alipay app.

Algorithms	Our Method				Prior Method [14]			
	Accuracy	Recall	Precision	F1	Accuracy	Recall	Precision	F1
**RF**	0.933	0.933	0.939	0.935	0.829	0.812	0.811	0.806
**AdaBoost**	**0.939** **(+0.120)**	**0.943** **(+0.142)**	**0.947** **(+0.138)**	**0.942** **(+0.139)**	0.819	0.801	0.809	0.803
**GBDT**	0.936	0.935	0.942	0.937	0.822	0.801	0.807	0.799
**XGBoost**	0.938	0.938	0.945	0.940	0.836	0.816	0.822	0.814

**Table 8:** Results of step identification within action on the WeChat app.

Algorithms	Our Method				Prior Method [14]			
	Accuracy	Recall	Precision	F1	Accuracy	Recall	Precision	F1
**RF**	0.961	0.957	0.962	0.957	0.957	0.954	0.956	0.942
**AdaBoost**	**0.970** **(+0.006)**	**0.968** **(+0.020)**	**0.973** **(+0.029)**	**0.969** **(+0.024)**	0.964	0.948	0.954	0.945
**GBDT**	0.960	0.958	0.964	0.959	0.957	0.939	0.950	0.939
**XGBoost**	0.967	0.965	0.968	0.965	0.963	0.947	0.962	0.947

**Table 9:** Results of UnionPay app identification.

Algorithms	Accuracy	Recall	Precision	F1
**RF**	0.980	0.978	0.998	0.987
**AdaBoost**	0.994	0.998	0.994	0.996
**GBDT**	0.978	0.980	0.994	0.987
**XGBoost**	0.994	0.995	0.997	0.996

**Table 10:** Results of action identification on the UnionPay app.

Algorithms	Accuracy	Recall	Precision	F1
**RF**	0.896	0.871	0.882	0.873
**AdaBoost**	0.893	0.872	0.880	0.870
**GBDT**	0.902	0.881	0.897	0.886
**XGBoost**	0.902	0.872	0.882	0.874

**Table 11:** Results of step identification within an action on the UnionPay app.

Algorithms	Accuracy	Recall	Precision	F1
**RF**	0.808	0.791	0.802	0.789
**AdaBoost**	0.796	0.786	0.794	0.782
**GBDT**	0.799	0.785	0.798	0.784
**XGBoost**	0.803	0.789	0.809	0.789
